# Protocol for *in vivo* DNA-RNA hybrid immunoprecipitation, sequencing, and analysis from frozen mouse tissues

**DOI:** 10.1016/j.xpro.2026.104654

**Published:** 2026-07-23

**Authors:** Hassan Massalha, Cameron J. Chee, Julia S.P. Mawer, Francesco Puzzo, Magdalena P. Crossley

**Affiliations:** 1 Cancer Research UK Cambridge Institute, University of Cambridge, Cambridge CB2 0RE, UK; 2 Telethon Institute of Genetics and Medicine, 80078 Pozzuoli, Italy

**Keywords:** Cell isolation, Genetics, Genomics, Sequencing, ChIPseq, Antibody, Chromatin immunoprecipitation, ChIP

## Abstract

DNA-RNA hybrids (R-loops) form transiently on the genome and regulate cellular homeostasis. They also influence genome-editing outcomes, highlighting their therapeutic potential. Here, we present a protocol for high-resolution mapping of DNA-RNA hybrids from frozen mouse tissues, including genomic DNA extraction and isolation of digested DNA-RNA hybrids using the hybrid-specific S9.6 antibody. We detail procedures for processing hybrids for whole-genome sequencing to generate R-loop profiles.

For details on the use of this protocol, please refer to Puzzo et al.^1^

## Highlights

• Procedure for genome-wide, high-resolution mapping of R-loops in frozen mouse tissues
• Steps for DNA-RNA immunoprecipitation (DRIP) using the S9.6 antibody
• Detailed workflow for next-generation sequencing library preparation
• Bioinformatics analysis pipeline for DRIP-seq analysis

## Abstract (editor highlights)

Publisher’s note: Undertaking any experimental protocol requires adherence to local institutional guidelines for laboratory safety and ethics.

## Graphical abstract

**Figure:**
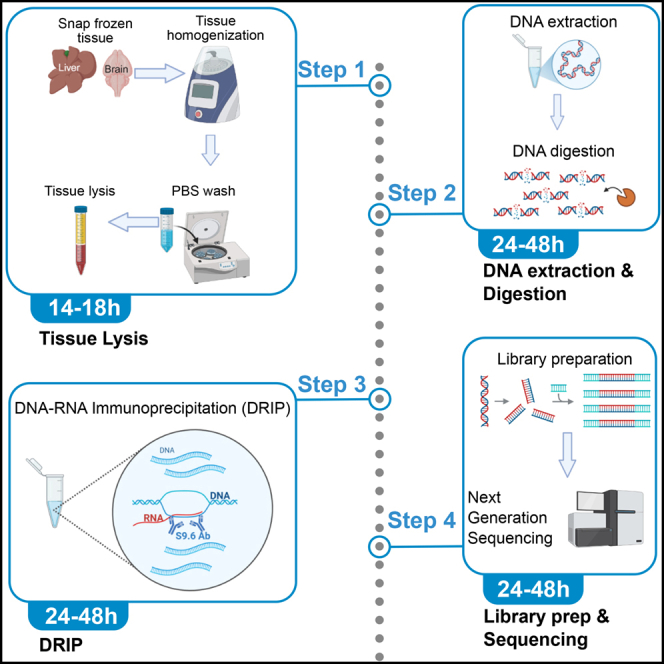

## Before you begin

This protocol describes genome-wide mapping of DNA–RNA hybrids (R-loops) directly from snap-frozen mammalian tissues by DNA–RNA hybrid immunoprecipitation followed by sequencing (DRIP-seq). The procedure has been validated using snap-frozen liver and brain from C57BL/6J mice and was applied in the context of adeno-associated virus (AAV)-mediated genome editing.^1^ The steps described below are illustrated for mouse liver; the same workflow has been successfully applied to mouse brain tissue and, with minor optimization, is expected to be applicable to other mammalian organs.

### Innovation

This protocol describes the first implementation of DRIP-seq directly on intact mammalian tissue. The previous DRIP-seq studies performed on tissue samples have generally relied on freshly isolated material followed by genomic DNA extraction from dissociated single cells. Our approach described herein enables the direct processing of snap-frozen tissue, allowing samples to be conveniently collected, stored, and analyzed without the requirement for immediate processing.^1^

By enabling genome-wide mapping of DNA-RNA hybrids (R-loops) directly from frozen tissues, this method expands R-loop profiling into physiologically relevant *in vivo* contexts beyond cultured cell lines, providing a practical framework for studying the distribution and potential functional roles of R-loops in animal models of disease. In addition, we provide bioinformatic workflows to directly compare matched tissue and cell-line DRIP-seq datasets, broadening the applicability of DRIP-seq to tissue-based studies and enabling systematic investigation of R-loop dynamics across diverse biological and pathological settings.

### Institutional permissions

Animal experiments described in the present protocol were conducted and approved by the Administrative Panel on Laboratory Animal Care of Stanford University relative to the publication Puzzo F., Crossley M.P., et al.^1^

### Tissue harvesting and preparation

The following procedure has been validated for snap-frozen organs (liver and brain) collected upon transcardiac perfusion.

1. Deeply anesthetize the mice.
2. Transcardially perfuse animals with room-temperature Dulbecco’s phosphate-buffered saline (DPBS).^2^^,^^3^
3. Transfer to 2 mL tubes, and immediately snap freeze on dry ice.

**CRITICAL:** Take extra caution when working with dry ice. Work in a well-ventilated workspace.

4. Store samples at −80°C until further use.

### Phase-separation gel preparation

The following phase separation gel protocol for making phase lock tubes was validated for use in DNA extraction, digestion and DNA-RNA hybrid immunoprecipitation.

**Alternatives:** Commercially available phase lock tubes have been discontinued, so homemade phase lock tubes have been provided as an alternative. While 15 mL tubes are no longer available, 2 mL phase lock tubes can still be found (https://www.lifesct.com/Phase-Lock-Gel).

5. Add High Vacuum Grease and 10% Silicon Dioxide (SiO_2_) to a high quality ziploc bag, then mix thoroughly using a combination of manual kneading and rolling (Figures 1A and 1B).

**Figure 1:**
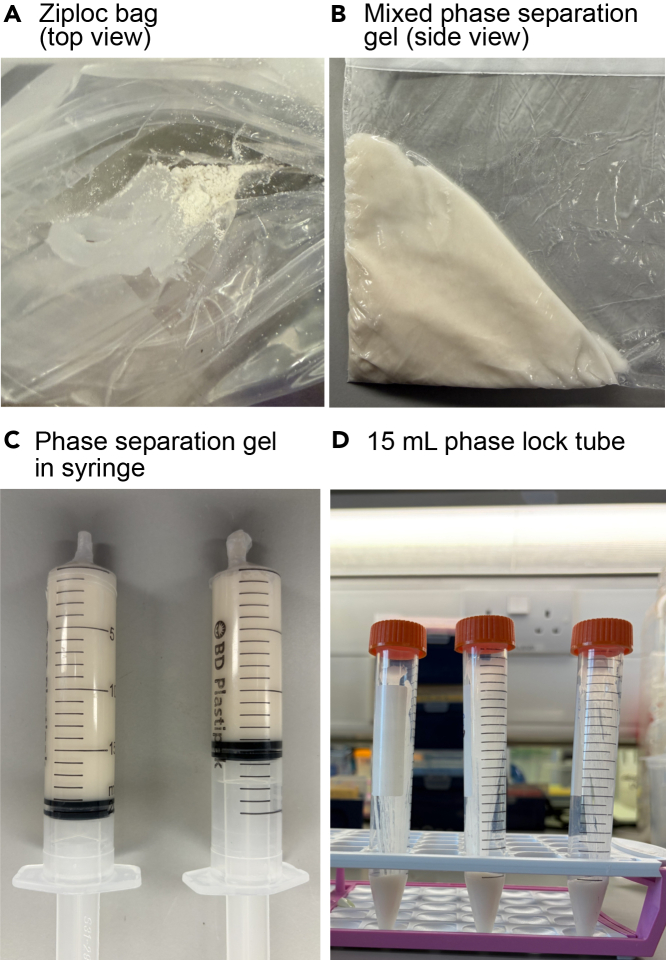
Preparation of phase lock tubes (A) 10% SiO_2_ vacuum grease before mixing. (B) 10% SiO_2_ vacuum grease mixed into a smooth paste. (C) Phase separation gel in syringes. (D) 15 mL phase lock tubes stored upright at 21-23°C.
6. Cut a corner of the plastic bag, dispense the phase separation gel into syringes and autoclave (Figure 1C**)**.
7. After cooling to 21–23°C, aliquot 1.5 mL of the gel into 15 mL tubes (100 μL into 2 mL tubes), spin down at 3000 × *g* for 1 min (16,000 × *g* for 2 mL tubes), and store upright at 21–23°C until needed (Figure 1D**)**.

## Key resources table

**Table:** 

REAGENT or RESOURCE	SOURCE	IDENTIFIER
**Antibodies**		

S9.6 monoclonal antibody (20 μg per 8 μg gDNA/reaction)	SIGMA, Millipore	MABE1095

**Chemicals, peptides, and recombinant proteins**		

Tris (1 M, pH 8)	Thermo Fisher	AM9855G
10X Tris-acetate-EDTA (TAE)	Sigma-Aldrich	T9650-1L
Dulbecco’s PBS (DPBS 1X)	Thermo Fisher, Gibco	14190–144
EDTA (500 mM)	Thermo Fisher	AM9260G
Sodium acetate (NaOAc; 3 M, pH 5.2)	Sigma-Aldrich	S7899-100ML
SDS (20% wt/vol)	Sigma-Aldrich	05030-500ML-F
Agarose I (Molecular Biology Grade)	Thermo Scientific	17850
Proteinase K	Thermo Fisher	25530049
Spermidine (0.1 M)	Sigma-Aldrich	05292-1ML-F
BSA (100X)	NEB	B9001
DNase/RNase free Water	Thermo Fisher	W4502
Phenol/Chloroform/isoamyl alcohol (25:24:1)	Thermo Fisher	15593031
High Vacuum Grease	Scientific Laboratory Supplies	Z273554
Silicon Dioxide, ∼99%, 0.5–10 μm (approx. 80% between 1-5 μm)	Scientific Laboratory Supplies	S5631-100G
Dynabeads protein G beads	Thermo Fisher	10003D
RNase H	NEB	M0297S
Glycogen	Thermo Fisher	R0561
NEBNext® End Repair Module	NEB	E6050S
Klenow Fragment (3'→5' exo-)	NEB	M0212S
IDT xGen™ Stubby Adapter and UDI Primers (Alternative: xGen Stubby Adapters can be purchased separately together with IDT xGen UDI primers)	IDT	10005976 (or 10005921)
AMPure XP beads	Beckman Coulter	A63880
Sodium phosphate, dibasic, anhydrous (HNa_2_O_4_P)	Thermo Fisher	S374-500
Sodium phosphate, monobasic (H_2_NaO_4_P)	Thermo Fisher	S397-500
Sodium chloride (NaCl; 5 M)	Sigma-Aldrich	SX0420-5
Bsp1407I	Thermo Fisher	FD0933
EcoRI	Thermo Fisher	FD0274
HindIII	Thermo Fisher	FD0504
SspI	Thermo Fisher	FD0774
XbaI	Thermo Fisher	FD0684
Ethyl alcohol, Pure	Sigma-Aldrich	459844
RINO Tubes, RNA Lysis Kit 50 pack (1.5 mL)	Next Advance	NA-NAVYR1-RNA
Sterile syringe filters 0.22 μm	Sigma-Aldrich	SLGPR33RS
50 mL Luer Lock syringes	Fisher Scientific	12339159
Quick Ligase	NEB	M2200S
Q5®-XT Hot Start High-Fidelity 2X Master Mix	NEB	M2499S
Sybr Green 2X mix	Thermo Fisher	4309155
MinElute PCR Purification Kit for PCR Cleanup	QiaGen	28004

**Experimental models: Organisms/strains**		

C57BL/6J mice (Species: Mus Musculus, 6-8 weeks-old male)	The Jackson Laboratory	000664

**Oligonucleotides**		

Alb#1 DRIP-qPCR FWD: GAGAGGAACCATTGCCACCT	N/A	N/A
Alb#1 DRIP-qPCR REV: CGATGGGCGATCTCACTCTT	N/A	N/A
Alb#2 DRIP-qPCR FWD: GCAGGGCTGAGAACGAGTTTA	N/A	N/A
Alb#2 DRIP-qPCR REV: AGCAAGTCTGCAGTTTGCTG	N/A	N/A
Alb#3 DRIP-qPCR FWD: CTCTTGCTGAGCTGGTGAAG	N/A	N/A
Alb#3 DRIP-qPCR REV: TCCAGAGAGCTACACCTGAC	N/A	N/A
Actb DRIP-qPCR FWD: TGCTCCCCGGGCTGTATT	N/A	N/A
Actb DRIP-qPCR REV: ACATAGGAGTCCTTCTGACCCATT	N/A	N/A
Eif5a DRIP-qPCR FWD: CCACTTACATCTGGCTGGAC	N/A	N/A
Eif5a DRIP-qPCR REV: CCTTGGGTCTCACTCATCC	N/A	N/A
ChrNeg DRIP-qPCR FWD: TTCCAACAAAGCAGCAAATG	N/A	N/A
ChrNeg DRIP-qPCR REV: GGGTCACCAGACCTGTTTTT	N/A	N/A

**Software and algorithms**		

Python v.3.11	N/A	https://www.python.org/downloads/release/python-31111/
Nextflow v.25.10.2	Di Tommaso et al.^4^	https://github.com/nextflow-io/nextflow
cutadapt v.5.2	Martin et al.^5^	https://cutadapt.readthedocs.io/en/stable/
Bowtie2 v.2.5.4	Langmead et al.^6^	https://github.com/BenLangmead/bowtie2?tab=readme-ov-file
SAMtools v.1.6	Danecek et al.^7^	https://www.htslib.org/
deepTools v.3.5.6	Ramírez et al.^8^	https://deeptools.readthedocs.io/en/latest/
pybedtools v0.12.0	Quinlan et al.^9^	https://daler.github.io/pybedtools/
Igv v.2.19.17	N/A	https://igv.org/
MACS2 v.2.2.9.1	N/A	https://anaconda.org/channels/bioconda/packages/macs2/overview

**Other**		

Tissues Homogenizer, Bullet Blender Storm Pro (1.5 mL screw and snap-cap tubes)	Next Advance	BT24M
Covaris Ultrasonicator	Covaris	Several models available
Agilent Bioanalyzer or Tapestation	Agilent Technologies	N/A
CFX384 Real-Time PCR system	Biorad	Several models available
Qubit Fluorometer	Thermo Fisher Scientific	Several models available

## Materials and equipment

**Table:** Phase separating gel

Reagent	Final concentration	Quantity or volume
High Vacuum Grease	N/A	5 g
Silicon Dioxide (∼99% 0.5–10 μm)	10% w/w	0.5 g
**Total**	–	**5.5 g**

Store at 21-23°C for up to 2 months.

**Table:** TE buffer

Reagent	Final concentration	Quantity or volume
Tris (1 M, pH 8)	10 mM	500 μL
EDTA (500 mM)	1 mM	100 μL
DNase/RNase-free water	–	49.4 mL
**Total**	–	**50 mL**

Filter the solution with a 0.22 μm filter and store it at 21–23°C for up to 1 month.

**Table:** Sodium phosphate 1 M pH 7

Reagent	Final concentration	Quantity or volume
Sodium phosphate, monobasic	2 M	39 mL
Sodium phosphate, dibasic, anhydrous	2 M	61 mL
DNase/RNase-free water	–	100 mL
**Total**	**1 M**	**200 mL**

If this solution crystallizes or precipitates, warm it up to resolubilize it.

The solution can be stored indefinitely at 21–23°C.

**Table:** 1% Agarose gel

Reagent	Final concentration	Quantity or volume
Agarose I	1% w/v	0.5 g
10X Tris-acetate-EDTA (TAE buffer)	1X	5 mL
DNase/RNase-free water	–	45 mL
**Total**	–	**50 mL**

We recommend preparing 50 mL of agarose gel; however, this can be scaled to any other volume as needed.

Mix the ingredients and heat until fully melted. Cool to 50–60°C, add DNA stain if needed, pour into the casting tray with a comb, and allow the gel to solidify. Do not store. Prepare fresh each time.

**Table:** 10× DRIP binding buffer

Reagent	Final concentration	Quantity or volume
Sodium phosphate, pH 7 (from Sodium phosphate table)	100 mM	2.5 mL
Sodium chloride (3 M, stock solution)	1.4 M	11.7 mL
Triton X-100	0.5% (vol/vol)	125 μL
DNase/RNase-free water	–	10.6 mL
**Total**	–	**25 mL**

Filter the solution with a 0.22 μm filter and store it at 21-23°C for up to 1 month.

**Table:** DRIP elution buffer

Reagent	Final concentration	Quantity or volume
Tris (1 M, pH 8)	50 mM	1.25 mL
EDTA (500 mM)	10 mM	500 μL
SDS (20% w/vol)	0.5% (vol/vol)	625 μL
DNase/RNase-free water	–	22.6 mL
**Total**	–	**25 mL**

Filter the solution with a 0.22 μm filter and store it at 21–23°C for up to 1 year.

## Step-by-step method details

1. We recommend using a minimum of two biological replicates (two animals) for each experimental condition.
2. All centrifugation steps are performed at 21–23°C, unless otherwise specified.

### Tissue lysis

**Timing: 14–18 h**

This procedure describes how to perform the lysis of the snap-frozen organ for the next step of DNA extraction.

3. Retrieve snap-frozen liver tissue (the mice must be cardiacally perfused with DPBS) from −80°C and allow it to thaw on ice for 30 min.
  ***Note:*** The cardiac perfusion of the animal will avoid and minimize contamination from blood cells.
  a. Transfer tissue into RINO tubes (2 to 3 tubes per liver).
    ***Note:*** If stored as a single frozen piece, section it on dry ice into fragments small enough to fit the tubes.
  b. Add 500 μL ice-cold sterile DPBS to each tube and homogenize using a Next Advance homogenizer for 5 min at speed 8.
  c. Pool all homogenates into a single 2 mL tube to ensure representation of the entire organ.
    ***Note:*** The homogenization of the whole liver is important to avoid zonal metabolic and transcriptional bias due to the partial organ homogenization.
4. Dilute 250 μL homogenate in 5 mL PBS and centrifuge at 300 × *g* for 5 min at 4°C.
  a. Repeat the wash twice for a total of 3 washes.
  b. Aliquot the homogenate and store at −80°C for up to one year for later DRIP studies or nucleic acid/protein extraction.
5. After the last wash, resuspend the final pellet containing the hepatic cells in 1.6 mL of TE Buffer.
6. Add 8 μL Proteinase K and 50 μL of 20% SDS.
7. Gently mix by inverting the tube 3 to 4 times.
8. Incubate the tube at 37°C for 12–16 h in a water bath or an incubator.

### DNA extraction and digestion

**Timing: 2–48 h**

This section describes the extraction of the genomic DNA from tissues gently lysed for 12-16 h. It also explains how to fragment the DNA by using a specific cocktail of restriction enzymes.

9. Centrifuge 15 mL homemade phase lock tubes (see the Phase separation gel preparation section) at 3,000 × *g* for 5 min.
  a. Transfer the lysate incubated for 12-16 h at 37°C into the 15 mL phase lock tube.
  b. Add an equal volume (1.6 mL) of phenol:chloroform:isoamyl alcohol (25:24:1).
  c. Invert 4-5 times and centrifuge at 1,500 × *g* for 5 min.

***Note:*** After this step, the supernatant should be clear. If not, spin for an additional 5 min.

10. Add a 1/10 volume of 3 M NaOAc, pH 5.2 (160 μL), and 2.5 volumes of 100% (vol/vol) ethanol (4 mL) to a new 15 mL tube.
  a. Pour in the DNA (the top aqueous phase) from the phase lock gel tube and mix the DNA and ethanol gently by inverting the tube until the DNA fully precipitates (visible by eye as a white precipitate).
  b. Wait for 10 min until all DNA is precipitated.
  c. Spool the DNA out of the mixture with a 1000 μL cut tip and transfer it to a clean 2 mL tube.

**CRITICAL:** Do not pellet DNA by centrifugation to minimize RNA contamination and preserve DNA-RNA hybrids.

11. Wash the DNA by adding 1 mL of 80% (vol/vol) ethanol freshly prepared, gently inverting the tube 2-3 times and letting it stand for 3 min.
  a. Carefully discard the supernatant; avoid pipetting the DNA.
  b. Repeat this step twice for a total of three washes.

**CRITICAL:** Very carefully remove as much ethanol as possible by pipetting after the last wash.

12. Allow to air dry completely while inverting tubes (to give a clear pellet).
  ***Note:*** This usually takes ∼10 min. If more cells are lysed this can take longer. Marking the location of the DNA on the tube can help to locate it once it has dried.
  a. Add 125 μL of TE buffer directly to the DNA pellet and carefully dislodge the cell pellet from the side of the tube with a pipette tip.
  b. Keep on ice for 20 min.
  c. Gently resuspend the DNA by pipetting two or three times with a 200 μL cut tip.
    **CRITICAL:** Avoid vortexing and overpipetting; maintaining the DNA viscosity will better preserve intact DNA-RNA hybrids.
13. Digest the entire solution (∼125 μL) of extracted genomic DNA using a cocktail of restriction enzymes: BsrGI (Bsp1407I), EcoRI, HindIII, SspI and XbaI (5 μL/enzyme in each reaction). Incubate for 12–16 h at 37°C.
  a. For 200 μL final reaction volume: 125 μL DNA + 5 μL × 5 enzymes (total 25 μL) + 20 μL of 10X FastDigest universal buffer (supplied by the vendor with the enzymes) + 27 μL DNase/RNase-free water + 1.5 μL BSA + 1.5 μL Spermidine.

**CRITICAL:** Spermidine should be added last at the proper concentration; excess spermidine can cause DNA precipitation.

***Note:*** After 12–16 h incubation, the viscosity of the mixture should have disappeared if the digestion is complete.

14. Spin a 2 mL phase lock tube (see the Phase separation gel preparation section) for 1 min at 16,000 × *g* to pellet the gel.
  a. Gently pipette the DNA from the previous step (200 μL) into the phase lock gel tube.
  b. Add 100 μL of DNase/RNase-free water and 1 volume (300 μL) of phenol/chloroform/isoamyl alcohol (25:24:1).
  c. Gently invert the tube 4 to 6 times and spin down at 16,000 × *g* for 10 min.
15. In a clean 1.5 mL tube, mix 1.5 μL of glycogen, 1/10 volume of 3 M NaOAc pH 5.2 (30 μL), and 2.5 volumes of 100% (vol/vol) ethanol (750 μL).
  a. Gently pipette in the DNA (top aqueous phase) from the phase lock gel tube and mix by inverting 4 to 6 times.
  b. Incubate for at least 2 h at −20°C (incubation for 12–16 h will enhance the DNA recovery) to increase precipitation yields.
16. Spin at 16,000 × *g* for 35 min at 4°C.
  a. Discard the supernatant.
  b. Add 200 μL of freshly made room-temperature 80% (vol/vol) ethanol.
  c. Spin for 10 min at 16,000 × *g* at 4°C and discard the supernatant.
17. Air dry the pellets for 10 min at 21–23°C until they become transparent, depending on the DNA concentration, and resuspend in 50 μL of TE buffer.
  a. Leave the tube on ice for 30-60 min and then gently resuspend by pipetting.
  b. Measure the concentration (OD260) of your DNA on a spectrophotometer. Typically, you should expect a concentration of at least ∼1 μg/μL.

***Note:*** To resuspend the DNA, do not vortex the tube or overpipette. Leave on ice longer or add an additional 50 μL TE buffer to help resuspension.

***Note:*** A specificity control is provided by including a sample that is pre-treated with RNase H to remove DNA-RNA hybrids from the mixture. Store the DNA that is not treated with RNase H at 4°C for 12–16 h before starting the IP the day after. (It can be stored for a few days at 4°C).

**Pause point:** The DNA can be stored at −80°C for up to a month.

18. Dilute 4–10 μg of digested DNA with 5–10 μL RNase H in 1X RNase H digestion buffer.

***Note:*** The final reaction volume should be at least 10 times the volume of RNase H enzyme added (i.e., 8 μg of DNA + 1X RNase H buffer + 8 μL RNase H + up to 80 μL of RNase/DNase-free water). Add the enzyme last to the mixture.

19. Incubate for 12–16 h at 37°C.

### DNA-RNA immunoprecipitation

**Timing: 24–48 h**

This section describes how to prepare the sample and perform the immunoprecipitation of the DNA-RNA hybrids (R-loops) using the S9.6 monoclonal antibody.

20. Samples treated with RNase H.
  a. Add 20 μL of RNase/DNase-free water to the RNase H solution up to a final volume of 100 μL.
  b. Add the sample to a 2 mL phase lock tube with 1 volume (100 μL) of Phenol:Chloroform:Isoamyl alcohol (25:24:1) and invert the tubes 4-5 times.
  c. Spin the phase lock tubes for 10 min at 16,000 × *g*.
  d. Add the top aqueous phase to a clean tube containing 1.5 μL of glycogen, 1/10 volume of 3 M NaOAc pH 5.2 (10 μL), and 2.5 volumes of 100% (vol/vol) ethanol (250 μL) and invert the tubes 4 to 5 times.
  e. Incubate for at least 2 h at −20°C or at least 1 h at −80°C to precipitate the DNA. Longer incubation times can be used, if practical.
  f. Spin at 16,000 x *g* for 35 min at 4°C.
  g. Discard the supernatant and add 200 μL of 80% (vol/vol) ethanol at 21–23°C.
  h. Spin for 10 min at 16,000 × *g* at 4°C and discard the supernatant.
  i. Air dry the pellets for 10 min at 21–23°C until they become transparent.
  j. Add 50 μl TE and leave the tubes on ice for 30 min to resuspend the pellets.
21. Dilute 8 μg DNA (both the RNase H-treated and the untreated samples) in a final volume of 500 μL TE.
  a. Save a 1/10 volume for each tube (50 μL) to use as input for qPCR. Store at −20°C.
22. Add 50 μL of 10X DRIP binding buffer and 20 μL of the S9.6 antibody to the 450 μL of diluted DNA.
  a. Incubate the tubes for 14–17 h at 4°C while gently inverting on a mini-tube rotator.
23. For each tube, wash 50 μL of the Dynabeads (equilibrate beads at 21-23°C for 10 min) with 700 μL of 1× DRIP binding buffer (1× DRIP binding buffer is made by diluting the 10X DRIP binding buffer in TE buffer) by inverting the tubes on a mini-tube rotator for 10 min at 21–23°C.
  a. Put the tubes onto a magnetic rack for 1 min and discard the supernatant.
  b. Repeat this step once for a total of 2 washes.
24. Add DNA:antibody complexes to prewashed beads, incubate 2 h at 4°C on a rotating shaker.
  a. Spin down beads using a bench-top microfuge to remove liquid from the lid and sides of the tube.
  b. Apply to the magnet and discard the supernatant.
25. Wash the beads:antibody complexes with 700 μl of 1X binding buffer.
  a. Invert 5–10 min at 21–23°C on a rotating shaker.
  b. Spin down for 10 min at 16,000 × *g* at 4°C to discard supernatant.
  c. Repeat twice for a total of 3 washes.
26. Add 250 μL of DRIP elution buffer and 8 μL of proteinase K (20 mg/mL) to the beads.
  a. Seal the tubes with Parafilm to avoid any leaking.
  b. Incubate with rotation at 55°C for 45 min in a temperature-controlled rotating oven.
27. Put the tubes onto a magnetic rack for 1 min.
  a. Meanwhile, spin 2 mL phase lock tubes for 1 min at 16,000 × *g* to pellet the gel.
  b. Transfer the supernatant of each tube to the 2 mL phase lock tube.
  c. Add 1 volume (250 μL) of phenol/chloroform/isoamyl alcohol (25:24:1).
  d. Invert gently 4 to 5 times.
  e. Spin down at 16,000 × *g* for 10 min at 21–23°C.
28. In a clean 1.5 mL tube, mix 1.5 μL of glycogen, 1/10 volume of 3 M NaOAc pH 5.2 (30 μL), and 2.5 volumes of 100% (vol/vol) ethanol (750 μL).
  a. Gently pipette in the DNA (top aqueous phase) from the phase lock tube.
  b. Mix by inverting 4 to 6 times.
  c. Incubate for at least 2 h (incubate for 12–16 h will increase the DNA:RNA hybrid recovery) at −20°C to increase precipitation yields.
29. Spin at 16,000 × *g* for 35 min at 4°C.
  a. Discard the supernatant.
  b. Add 200 μL of 80% (vol/vol) ethanol at 21–23°C.
  c. Spin for 10 min at 16,000 × *g* at 4°C and discard the supernatant.
30. Air-dry the pellets for 10–15 min.
  a. Add 50 μL of TE buffer to each tube.
  b. Leave the tubes on ice for 15–30 min and gently resuspend.

**Pause point:** The immunoprecipitated DNA:RNA hybrids can be stored at −80°C for up to six months.

31. Check the DRIP efficiency by qPCR.
  a. Dilute samples 1:5 in RNase/DNase-free water and use 3 μL for qPCR.

***Note:*** Before proceeding, it is advised to make sure that the DRIP procedure worked. To this end, use two negative and two to three positive loci and measure their immunoprecipitation as a fraction of input DNA. The RNase H-treated sample should lead to very low yields (>90% reduction in DRIP efficiency).

***Note:*** To calculate the percentage of input, apply the following formula for each locus: % input = 100∗2ˆ(Ct input(corrected) - Ct DRIPed DNA), where Ct input(corrected) = (Ct input - log2(10)) (we subtract log2(10) because the input represents 1/10th of the immunoprecipitated DNA).

### Library preparation

**Timing: 3–4 h**

This procedure describes the method to prepare libraries for the DRIP sequencing and their quality control using Bioanalyzer instrumentation.

32. Sonicate the DNA from step 30 to achieve a peak fragment size of ∼300 base pairs (bp). For the purposes of this protocol, a Covaris S220 with Covaris microtubes (Fisher, 520045) is used with the following sonication parameters: Intensity: 4; C/B: 200; DF: 10%; Time: 30 s.
  a. Load 5 μL of sheared DNA on a 1% agarose gel to verify the DNA has been homogeneously fragmented to a peak fragment size of 300–400 bp.
33. End repair the sheared DNA.
  a. Add 2.5 μL NEBNext end Repair enzyme + 5 μL NEB 10X buffer + 2 μL ATP to the 40.5 μL DNA for a total volume of 50 μL into 500 μL tubes.
  b. Pipet up and down to mix and incubate at 21–23°C for 30 min.
34. Clean up with MinElute QIAGEN Column.
  a. Add 250 μL of buffer PB.
  b. Add 10 μL of 3 M Sodium Acetate.
  c. Add 34 μL DNase/RNase-free water; wait 1 min before spinning to elute the sample.
35. A-tailing.
  a. Dilute dATP to 1:100 (from 100 mM stock).
  b. Add 5 μL NEB buffer 2 + 10 μL 1 mM dATP + 1 μL NEB Klenow Fragment (3'→5' exo-) to the 34 μL DNA for a total volume of 50 μL.
  c. Mix and incubate for 30 min at 37°C (in an incubator).

***Note:*** Use nuclease-free PCR tubes for this step.

36. Clean up with MinElute QIAGEN Column.
  a. Add 250 μL of buffer PB.
  b. Add 10 μL of 3 M Sodium Acetate.
  c. Elute in 12 μL, wait 1 min before spin elution.
37. Ligate adapters.
  a. To 12 μL A-tailed DNA (∼20-50 ng), add 2.0 μL xGen Stubby Adapter (3.0 μM, 1:5 diluted in RNase/DNase-free water) + 15 μL 2X NEB Quick Ligase Reaction Buffer + 1 μL NEB Quick Ligase for a total volume of 30 μL.
  b. Incubate at 21–23°C for 15 min.

***Note:*** Use nuclease-free PCR tubes for this step.

***Alternatives:*** This protocol was originally published using Illumina Prep2Seq indexed adapters. These are no longer commercially available, and we provide an alternative with Y-type adapters (xGen Stubby Adapters) without indexes, followed by UDI index addition at the PCR step. Library preparation can also be performed using a single kit for the whole process (for example NEBNext Ultra II DNA library kit). The modular library preparation workflow provided here is a more flexible and cheaper alternative.

***Note:*** For lower DNA input amounts (10–20 ng), using 2.0 μL of 1.5 μM adapters may be more appropriate and result in fewer adapter dimers in the libraries (see expected outcomes).

38. Do a one-sided size selection with Ampure beads to remove small fragments.
  a. Equilibrate Ampure beads to 21–23°C.
  b. Add 70 μL of DRIP elution buffer (EB) buffer to the 30 μL of DNA.
  c. Add 100 μL of Ampure beads, vortex, spin down quickly, and incubate for 5 min at 21–23°C.
  d. Apply to the magnet for 2 min or until the liquid clears completely and *discard the supernatant.*
  e. Wash 2 times with 200 μL fresh 80% EtOH (important to be made fresh), and dry for 5 min until the beads start “cracking.”
  f. Add 11 μL EB buffer, vortex and incubate for 5 min at 21–23°C.
  g. Apply to the magnet, wait for 2 min or until the liquid clears completely, and *take the supernatant.*
  h. Repeat 1:1 Ratio with Ampure beads.
  i. Add 90 μL of EB buffer to 10 μL of DNA (from previous Step).
  j. Add 100 μL of Ampure beads, vortex and spin down quickly, and incubate for 5 min.
  k. Apply to the magnet, wait for 2 min or until the liquid clears completely, and *discard the supernatant.*
  l. Wash 2 times with fresh 80% EtOH (important to be made fresh), and dry for 5 min until the beads start “cracking.”
  m. Add 11 μL EB, vortex, and incubate for 5 min at 21–23°C.
  n. Apply to the magnet, wait for 2 min or until the liquid clears completely, and *take the supernatant.*
39. Run qPCR to determine the number of PCR cycles needed to amplify the libraries.
  a. qPCR reaction setup: 2 μL of adapter-ligated DNA (can also take 1 μL DNA dilute 1:10 and do three replicates per sample, then subtract 3 cycles from the fitpoint to account for DNA dilution) + 0.5 μL xGen UDI Primer Pair + 7.5 μL of Sybr Green Master Mix + 5 μL of RNase/DNase-free water for a total volume of 15 μL per reaction. Use fresh Master Mix for each run.

***Note:*** The qPCR is run in a 384-well plate. However, the reaction volume can be scaled-up for a 96-well plate.

***Note:*** The fitpoint (**X**) gives you the number of cycles to use for the PCR (usually between 5 to 15) (Figure 2).

40. Library amplification PCR
  a. Prepare PCR reaction master mix following the amount specified in Table 1.

**Table 1:** PCR reaction master mix

Reagent	Amount (μL)
DNA template - adapter-ligated DNA (half of your available DNA)	5
DNA Polymerase - Q5-XT Hot Start High-Fidelity 2X Master Mix	15
Primer Mix - μL xGen UDI Primer Pair (20 μM)	1
RNase/DNase-free water	9
**Total**	**30**

Note: Prepare fresh each time.
  b. Record the UDI ID for the adapters used as this is required for demultiplexing and assigning samples.
  c. Run PCR program following the conditions in Table 2.

**Table 2:** PCR cycling conditions

Steps	Temperature	Time	Cycles
Initial Denaturation	98°C	30 s	1
Denaturation	98°C	10 s	X cycles (to be determined by qPCR in step 39)
Annealing	60°C	30 s	–
Extension	72°C	30 s	–
Final extension	72°C	5 min	1
Hold	4°C	Infinite

***Note:*** For NovaSeq and other patterned flow-cell instruments, unique dual indexes are recommended to minimise index hopping and sample misassignment.

**Figure 3:**
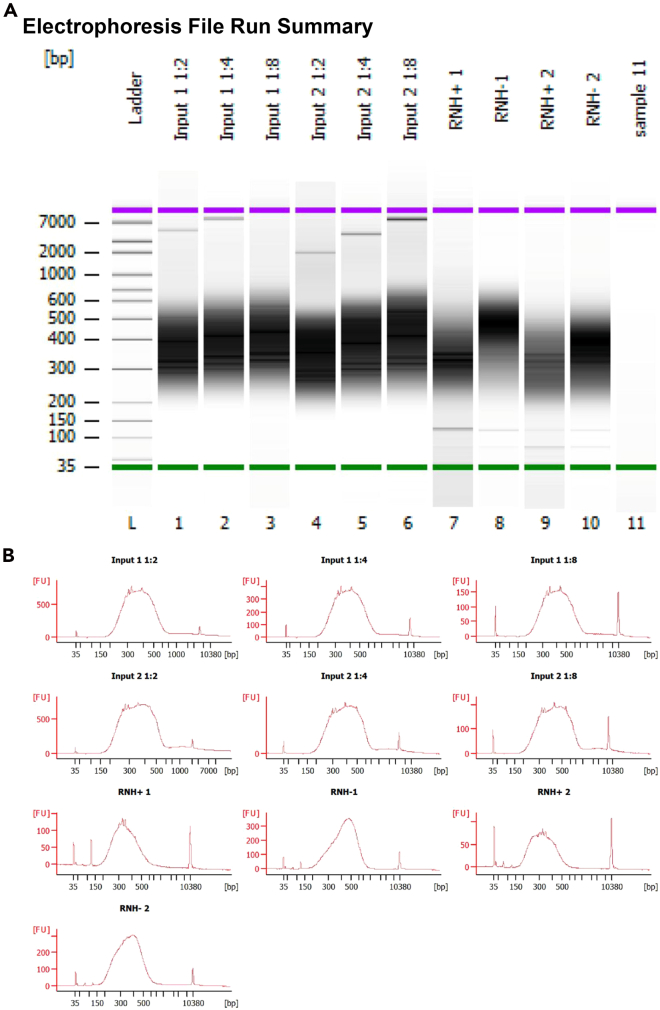
Library quality control Analysis of the PCR-amplified libraries by Bioanalyzer. (A) Digital gel and (B) electropherogram of capillary electrophoresis. Molecular sizes are indicated in base pairs (bp). Quality control data are shown for sequencing libraries published in Puzzo, Crossley et al.^1^
  m. Sequence the libraries using paired-end sequencing with a read length of 150 bp on an Illumina-compatible platform (e.g., NovaSeq, NextSeq).

***Note:*** To obtain robust sequencing results we recommend having a minimum of 30 million paired end reads per sample, with a target of 30–50 million.

41. Post-PCR two-sided library size selection. To each 30 μL of amplified DNA.
  a. Add 70 μL of EB buffer.
  b. Add 65 μL of Ampure beads.
  c. Vortex, spin down quickly and incubate for 5 min at 21–23°C.
  d. Apply to the magnet, wait for 2 min or until the liquid clears completely and take *the supernatant* into a fresh tube.
  e. Do a 1:1 ratio in the remaining supernatant.
  f. Add 100 μL EB (200 μL total: 30 +70 +100).
  g. Add 135 μL of beads slurry, vortex, spin down quickly and incubate for 5 min at 21–23°C.
  h. Apply to the magnet, wait for 2 min or until the liquid clears completely and *discard the supernatant.*
  i. Wash two times with fresh 80% EtOH, and dry for ∼5 min (beads should start “cracking”).
  j. Add 11 μL EB buffer, vortex and incubate for 5 min at 21–23°C.
  k. Apply to the magnet, wait for 2 min or until the liquid clears completely and *take the supernatant.*
  l. Quality control of the libraries are carried out on a Agilent Technologies BioAnalyzer or Tapestation (see expected results in Figure 3).

**Figure 2:**
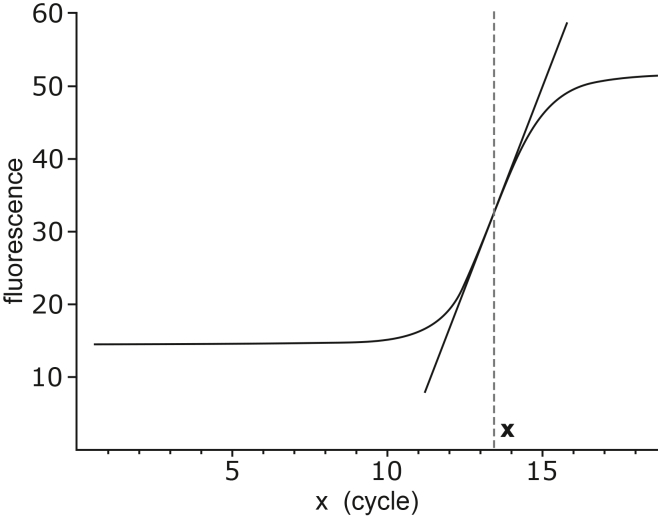
Library preparation overview and calculation of fitting point for library amplification Schematic of qPCR curve analysis to calculate the number of cycles for the library amplification by PCR. X indicates the number of PCR cycles required.

### DRIP-sequencing analysis

**Timing: 2 h**

42. Running the DRIP-seq bioinformatics analysis pipeline.
  The analysis code is developed openly on GitHub at https://github.com/MCrossleyLab/DRIP-seq_STAR_Protocol and the exact version corresponding to this manuscript is archived on Zenodo at https://doi.org/10.5281/zenodo.20343896.
  a. Prepare the input files. Populate the samplesheet.csv file with all relevant sample information, including: sample_id, fastq1, fastq2, species, treatment, replicate, tissue, and condition. Use absolute paths. Make sure that paths are accessible, and that file names are correct.
  b. Configure pipeline parameters. Complete the required fields in the pipeline_parameters.yml file, including the correct adapter sequences. Double-check that all directory paths, reference genome locations, and other parameters accurately reflect your analysis setup.
  c. Replace the adapter sequence based on the used kit (defult: IDT xGen™ Stubby Adapter).
  d. Set up the computing environment. The pipeline is written in Nextflow and can run either on a personal workstation or your institute’s computing cluster. Follow GitHub instructions to install Nextflow and all software dependencies.
    ***Note:*** If using a cluster, ensure that the architecture and executor configuration in the nextflow.config file match your cluster’s current setup.
  e. Run the pipeline by following the step-by-step instructions on the project’s GitHub page.
  f. Monitor progress. Nextflow automatically logs pipeline progress and organizes intermediate files. If errors occur, refer to the log files or the troubleshooting section below.
  g. Locate the output files at the following path outdir_path/outdir_name provided in the pipeline_parameters.yml file. This folder will contain subdirectories for different types of results.
43. QC assessment.
  a. Browse the log files in bowtie2_logs folder, as part of the output directories.
  b. Check the number of aligned reads, and the overall alignment rate as an initial QC assessment.

***Note:*** Outputs will depend on the sample type (ip vs input). A minimum of ∼30 million aligned reads per sample and a minimum of 90% read alignment in all samples are the expected numbers.

***Note:*** Check the nf_reports to optimize resource usage and execution time for each step of the pipeline. The report files (execution_report.html and timeline.html) are in outdir_path/outdir_name/nf_reports/.

44. Exploring output files.
  a. View normalized signal tracks stored at normalized_BigWig_files within your output directory. These files show CPM (Counts-Per-Million) normalized read coverage across the genome.
  b. Visualize results in IGV. Use the free Integrative Genomics Viewer (IGV) to explore the BigWig files. IGV allows you to compare tracks, inspect coverage, and visualize signal patterns.

***Note:*** Ensure IGV is installed and running on your machine before opening files. Download and follow installation instructions found at https://igv.org/.

45. Setting a contrast list.
  a. Decide on your replicates what to include in the contrast list.
  b. Set a contrast list composed of sample id (as in the samplesheet.csv) in the pipeline_parameters.yml file to perform peak calling.
    i. Follow the following structure: contrast_name: [[ip_sample1, ip_sample2, .], [in_sample1, in_sample2, ...]].
    ii. Expand the contrast list as needed by starting new line following the above structure.
  c. rerun the pipeline after modifying the pipeline_parameters.yml file. Nextflow will update the outputs accordingly.
46. Downstream analysis.
  a. Access additional output files. All BAM files and other pipeline outputs can be found in the same outdir_path/outdir_name directory.
  b. Perform further analyses. Follow the published guidelines in Puzzo F, Crossley MP et al^1^ for recommended downstream analysis steps and interpretation of DRIP-seq results.
  c. Peak files can then be further used for downstream differential analysis. DESeq2 can be used for peak discovery statistics.
  d. To analyze genome level features, we recommend using DeepTools (listed in the Software and algorithms table) to produce metaplots, mainly using the computeMatrix package.

***Note:*** The computational pipeline can be adapted to user specific analysis by changing the input fastq files. Likewise, we introduce the modular construct groups for peak calling step. The user can make advantage of this feature by customizing the test groups for downstream analysis.

## Expected outcomes

**Figure 4:**
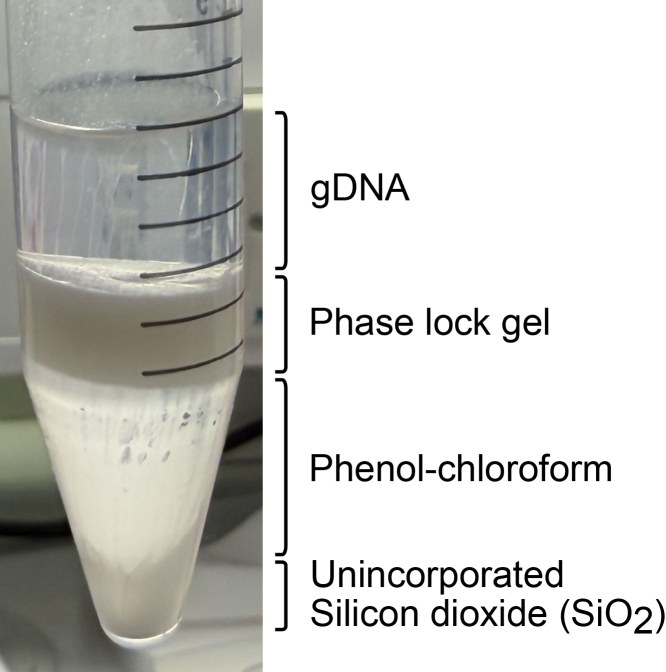
Expected phase lock tube separation of genomic DNA Phase separation in 15 mL phase lock tubes (following step 6). Aqueous phase containing genomic DNA separates above the phase separation gel.

After tissue lysis, genomic DNA is isolated via phenol:chloroform extraction and can be collected in the aqueous phase of homemade phase lock tubes, as described in Step 6. Successful phase separation is shown in Figure 4. Samples are fragmented by digestion with a cocktail of restriction enzymes, and DNA-RNA hybrids are immunoprecipitated using the hybrid-specific S9.6 monoclonal antibody. Our protocol results in libraries with a peak of 200-500 bp in fragment length, which are ready for DRIP sequencing (Figure 3).

A successful run of the analysis pipeline should take ∼1h:40min (using the configurations in the nextflow.config file). Below is a snapshot of a completed run log, initiated directly from the GitHub repository.

(DRIP-seq_STAR) [massal01@clust1-sub-2 ∼]$ nextflow run MCrossleyLab/DRIP-seq_STAR_Protocol \

> -profile slurmc \

> -with-conda \

> -resume \

> -params-file ∼/.nextflow/assets/MCrossleyLab/DRIP-seq_STAR_Protocol/pipeline_parameters.yml

N E X T F L O W ∼ version 25.10.2

Launching `https://github.com/MCrossleyLab/DRIP-seq_STAR_Protocol` [big_swanson] DSL2 - revision: d77c52406a [main]

[INFO] using samplesheet: /Users/massal01/.nextflow/assets/MCrossleyLab/DRIP-seq_STAR_Protocol/samplesheet.csv

[INFO] using genome_path: /Users/massal01/Genomics/indexes

[INFO] writing output to: /mnt/scratche/slow/mclab/massal01/DRIP-seq_STAR_Protocol/results_GitHub

[INFO] starting processes.

executor > slurm (110).

[e4/ed3adc] bowtie_reads:CUTADAPT_ADAPTER_TRIM (SRR28361288) [100%] 12 of 12 ✔

[8c/1cc872] bowtie_reads:BOWTIE2_ALIGN (SRR28361288) [100%] 12 of 12 ✔

[b7/170811] markdup_bams:SAMTOOLS_COLLATE (SRR28361288) [100%] 12 of 12 ✔

[75/b90d11] markdup_bams:SAMTOOLS_FIXMATE (SRR28361288) [100%] 12 of 12 ✔

[02/f86f66] markdup_bams:SAMTOOLS_SORT (SRR28361288) [100%] 12 of 12 ✔

[03/f80b4a] markdup_bams:SAMTOOLS_MARKDUP_INDEX (SRR28361288) [100%] 12 of 12 ✔

[3f/985f24] markdup_bams:SAMTOOLS_FLAGSTAT (SRR28361288) [100%] 12 of 12 ✔

[3d/42df95] bam_to_bigWig:COUNT_READS_RPM (SRR28361288-[mm10]-ip.RNAseH1-rep1) [100%] 12 of 12 ✔

[24/1a9334] bam_to_bigWig:BAMCOVERAGE (SRR28361288-mm10-ip.RNAseH1-rep1) [100%] 12 of 12 ✔

[e4/50d378] peak_calling:MACS2_CALLPEAK (hepa_ip_vs_in) [100%] 2 of 2 ✔

Completed at: 05-May-2026 23:00:38

Duration : 1h 38m 42s

CPU hours : 89.3

Succeeded : 110

**Figure 5:**
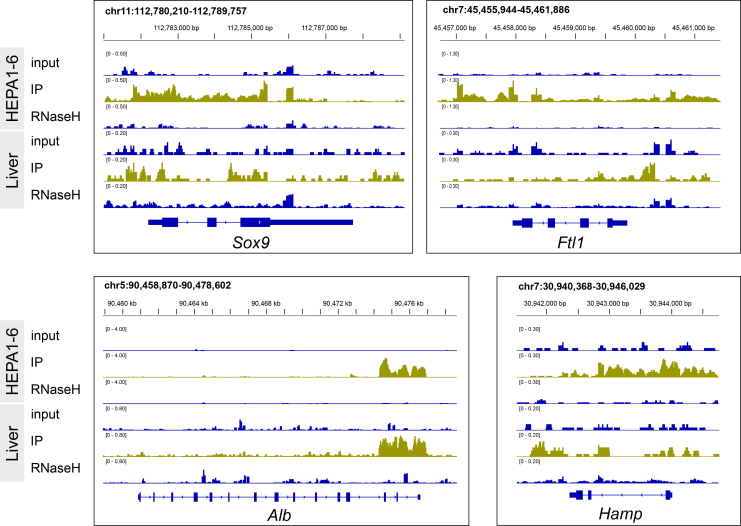
Genome browser tracks of normalized BigWig files from the bioinformatic pipeline BigWig tracks for 4 representative genes (*Sox9, Ftl1, Alb, Hamp*) are shown as IGV browser snapshots. Tissue identities and sample treatments (input, IP, RNase H) are indicated on the left. IP tracks are highlighted in green.

Following successful sequencing of libraries and alignment to the mouse genome, browser tracks of DRIP-seq signal can be viewed using IGV (Figure 5). Expect DRIP-seq signal to be robust over genes, including promoters and gene terminators, especially those that are highly expressed in liver (including *Sox9, Alb, Ftl1, Hamp)* (Figure 5). IP samples should show enrichment above input samples. Samples pre-treated with RNase H to remove DNA-RNA hybrids are expected to show reduced DRIP-seq signal genome-wide.

## Limitations

One limitation of this protocol is that snap-frozen tissue is processed in bulk. In the liver, DRIP profiles were comparable whether hepatocytes were isolated from the whole organ prior to genomic DNA extraction or the tissue was processed directly after snap-freezing^1^, consistent with the predominantly hepatocytic composition of this organ. Although whole brain tissue can also yield robust DRIP signal,^1^ processing bulk material precludes resolution of cell type-specific R-loop landscapes (for example, astrocytes or neurons versus oligodendrocytes). This protocol is therefore most appropriate for DRIP-seq at the whole-tissue level rather than for defining DNA-RNA hybrid formation in discrete cell populations within heterogeneous tissues. While we have validated the procedure using frozen mouse liver and brain, additional optimization and adaptation will likely be required to enable R-loop mapping in other mammalian tissues.

## Troubleshooting

### Problem 1

Incomplete liver homogenization (Tissue lysis, Step 3).

Liver tissue homogenization may be inconsistent depending on the homogenization system employed. Variability has been observed across instruments, particularly when using bead-based systems (e.g., Next Advance Homogenizer with RINO tubes containing metal beads). In such cases, differences in tissue composition among samples, such as varying degrees of fibrosis or lipid accumulation, can affect homogenization efficiency. Fibrotic or lipid-rich livers may exhibit increased resistance to mechanical disruption, resulting in incomplete homogenization.

### Potential solution

To improve homogenization efficiency, samples may be subjected to additional homogenization cycles until no visible tissue fragments remain. Alternatively, increasing the homogenization intensity (e.g., speed or duration) may enhance tissue disruption. Pre-processing steps may also improve outcomes; liver tissue can be subdivided into smaller fragments at the time of collection or prior to snap-freezing, thereby facilitating more uniform and efficient homogenization during subsequent processing.

### Problem 2

DNA fragmentation following centrifugation of the homogenate (Tissue lysis, Step 4).

Significant DNA fragmentation may be observed following centrifugation of the tissue homogenate and subsequent resuspension in TE buffer. One contributing factor is insufficient washing of the homogenate with DPBS, which may result in the persistence of cellular debris, including lysed cells and subcellular components. This is typically indicated by a cloudy supernatant after centrifugation. The presence of such contaminants can adversely affect DNA integrity and compromise downstream extraction and analysis.

### Potential solution

To minimize DNA fragmentation and improve sample quality, the homogenate should be thoroughly washed with DPBS. If the supernatant remains cloudy after the two wash steps, additional washing cycles are recommended. During each wash, the supernatant should be carefully aspirated and replaced with fresh DPBS until it becomes visually clear and a well-defined pellet is observed at the bottom of the tube. This ensures effective removal of residual debris prior to DNA extraction.

### Problem 3

Incomplete phase separation in homemade phase lock tubes (DNA extraction and digestion, Step 9).

Heavy density phase lock tubes are approximated by supplementing the phase separating gel with silicon dioxide. If they are not made properly, the aqueous phase will migrate below the phase lock.

### Potential solution

The gel is supplemented with at least 10% silicon dioxide (SiO_2_), but due to variance in particle size, this may be insufficient for good phase separation. Increasing to 15% SiO_2_ may help offset this difference. Alternatively, SiO_2_ can be size fractionated before adding it to the gel. To do so, suspend 10 g SiO_2_ in RNase/DNase-free water and let the larger particles settle for 2 h. Transfer the supernatant to a new tube, then centrifuge to collect remaining SiO_2_. Dry before use.

### Problem 4

Insufficient genomic DNA recovered from spooling (DNA extraction and digestion, Step 10).

Genomic DNA may be incompletely precipitated or lost due to mechanical shearing.

### Potential solution

Invert the tube thoroughly to ensure all genomic DNA is precipitated. Use gentle inversion to avoid breaking up the DNA into fragments, which can otherwise be lost in subsequent wash steps (Step 11).

### Problem 5

Low yield after immunoprecipitation due to ethanol contamination (DNA extraction and digestion, Step 19).

If pellets are insufficiently dried, contaminating ethanol can reduce restriction digestion and immunoprecipitation efficiency.

### Potential solution

Remove remaining ethanol with a P10 tip. Allow the pellet to air dry and only proceed when all ethanol drops have disappeared and the pellet is transparent.

### Problem 6

DRIP-seq pipeline is not working (DRIP-Sequencing analysis, Step 42).

### Potential solution

Verify that all steps outlined in the GitHub repository have been followed carefully. We highly recommend reviewing the error messages to identify the specific issue and guide troubleshooting. Ensure that the Nextflow run command is executed from the correct environment (see instructions in GitHub). Check that all file paths specified in the pipeline_parameters.yml and samplesheet.csv files are accurate and absolute.

### Problem 7

DRIP-seq pipeline is too slow (DRIP-Sequencing analysis, Step 42).

### Potential solution

The DRIP-seq pipeline is built in Nextflow, which is designed to parallelize tasks and improve computational efficiency. To achieve optimal performance, it is strongly recommended to run the analysis on a high-performance computing cluster rather than a local machine. Before doing so, check that the cluster settings in the nextflow.config file are correctly configured and match your cluster’s current architecture. This setup only needs to be done once, and the pipeline will automatically apply the configuration in future runs. Additionally, we recommend viewing the pipeline run reports (execution_report.html and timeline.html) as they contain run resource use and can help with optimizing the requested resource for each step of the pipeline. For additional guidance, you can refer to the official Nextflow documentation at https://www.nextflow.io/docs/latest/executor.html or consult your IT team for support.

## Resource availability

### Lead contact

Further information and requests for resources and reagents should be directed to and will be fulfilled by the lead contact, Magdalena P. Crossley (mpk33@cam.ac.uk).

### Technical contact

Technical questions on executing this protocol should be directed to and will be answered by the technical contacts, Francesco Puzzo (experimental questions; f.puzzo@tigem.it) and Hassan Massalha (bioinformatics questions; hm670@cam.ac.uk).

### Materials availability

This study did not generate new unique reagents.

### Data and code availability

Original DRIP-seq data files reported in this *STAR Protocols* are available in Puzzo et al.^1^ under GEO Database: GSE261759. The analysis code used in this study is archived on Zenodo at https://doi.org/10.5281/zenodo.20343896 and is openly available under the license specified in the repository.

## Acknowledgments

We thank members of the Crossley lab for helpful discussions. This work was supported by the 10.13039/501100022011Cancer Research UK Cambridge Institute Core Award (SEBINT-2024/100003) to M.P.C. We also thank Matthew Eldridge and Ashley Sawle from the Cancer Research UK Cambridge Institute Bioinformatics Core facility for guidance and support in deploying the analysis pipeline on GitHub.

## Author contributions

F.P. and M.P.C. conceptualized the study. F.P. and C.J.C. designed and conducted the experiments. F.P. conducted *in vivo* DRIP-sequencing experiments. C.J.C. developed and optimized phase lock gel separation. H.M. developed and implemented the Nextflow pipeline and performed bioinformatics analysis. All authors wrote the manuscript. M.P.C. provided supervision and funding.

## Declaration of interests

The authors declare no competing interests.
